# How the Dorsolateral Prefrontal Cortex Controls Affective Processing in Absence of Visual Awareness – Insights From a Combined EEG-rTMS Study

**DOI:** 10.3389/fnhum.2018.00412

**Published:** 2018-10-16

**Authors:** Kati Keuper, Esslin L. Terrighena, Chetwyn C. H. Chan, Markus Junghoefer, Tatia M. C. Lee

**Affiliations:** ^1^Institute of Biomagnetism and Biosignalanalysis, University Hospital Münster, University of Münster, Münster, Germany; ^2^Otto Creutzfeldt Center for Cognitive and Behavioral Neuroscience, University of Münster, Münster, Germany; ^3^Laboratory of Neuropsychology, The University of Hong Kong, Pokfulam, Hong Kong; ^4^Laboratory of Social Cognitive Affective Neuroscience, The University of Hong Kong, Pokfulam, Hong Kong; ^5^Applied Cognitive Neuroscience Laboratory, Department of Rehabilitation Sciences, The Hong Kong Polytechnic University, Kowloon, Hong Kong; ^6^The State Key Laboratory of Brain and Cognitive Sciences, The University of Hong Kong, Pokfulam, Hong Kong; ^7^Institute of Clinical Neuropsychology, The University of Hong Kong, Pokfulam, Hong Kong

**Keywords:** EEG, rTMS, emotion perception, awareness, ERP, dlPFC

## Abstract

The dorsolateral prefrontal cortex (DLPFC) plays a key role in the modulation of affective processing. However, its specific role in the regulation of neurocognitive processes underlying the interplay of affective perception and visual awareness has remained largely unclear. Using a mixed factorial design, this study investigated effects of inhibitory continuous theta-burst stimulation (cTBS) of the right DLPFC (rDLPFC) compared to an Active Control condition on behavioral (*N* = 48) and electroencephalographic (*N* = 38) correlates of affective processing in healthy Chinese participants. Event-related potentials (ERPs) in response to passively viewed subliminal and supraliminal negative and neutral natural scenes were recorded before and after cTBS application. We applied minimum-norm approaches to estimate the corresponding neuronal sources. On a behavioral level, we found evidence for reduced emotional interference by, and less negative and aroused ratings of negative supraliminal stimuli following rDLPFC inhibition. We found no evidence for stimulation effects on self-reported mood or the behavioral discrimination of subliminal stimuli. On a neurophysiological level, rDLPFC inhibition relatively enhanced occipito-parietal brain activity for both subliminal and supraliminal negative compared to neutral images (112–268 ms; 320–380 ms). The early onset and localization of these effects suggests that rDLPFC inhibition boosts automatic processes of “emotional attention” independently of visual awareness. Further, our study reveals the first available evidence for a differential influence of rDLPFC inhibition on subliminal versus supraliminal neural emotion processing. Explicitly, our findings indicate that rDLPFC inhibition selectively enhances late (292–360 ms) activity in response to supraliminal negative images. We tentatively suggest that this differential frontal activity likely reflects enhanced awareness-dependent down-regulation of negative scene processing, eventually leading to facilitated disengagement from and less negative and aroused evaluations of negative supraliminal stimuli.

## Introduction

The human brain preferentially processes negative information, even if this information is presented in absence of visual awareness (e.g., Bayle et al., 2009; for an fMRI meta-analysis, see Meneguzzo et al., 2014). This is reflected in faster detection and preferential processing of negative images, even if participants cannot report image contents above chance (i.e., subliminal exposure; Fazio, 2001; Spruyt et al., 2002; Winkielman and Berridge, 2004; Winkielman et al., 2005; Kiss and Eimer, 2008; Sabatini et al., 2009; Meneguzzo et al., 2014). A key brain structure that affects automatic and elaborate affective perception and modulates corresponding motivational responses is the dorsolateral prefrontal cortex (DLPFC) (Grisaru et al., 2001; Spielberg et al., 2008; De Raedt et al., 2010; Leyman et al., 2011). It plays a crucial role in top-down analysis and regulation of affective processing (Banks et al., 2007; Goldin et al., 2008; Notzon et al., 2017; Wager et al., 2008; Zwanzger et al., 2014). Valence-specific characteristics of such inhibitory control processes might be influenced by hemispheric asymmetries in prefrontal brain functioning (Grimshaw and Carmel, 2014). Such asymmetries likely modulate withdrawal and approach-related affect, whereby right hemispheric dominance has been linked to negative, withdrawal-related processing (for reviews, see Davidson, 1992a,b; Harmon-Jones et al., 2010; Grimshaw and Carmel, 2014).

In the current study, we investigated how the processing of subliminal and supraliminal negative, withdrawal-associated stimuli is affected by right DLPFC (rDLPFC) inhibition via transcranial magnetic stimulation (TMS). By combining TMS with electroencephalographic measures, we specifically investigate the spatiotemporal interplay of rDLPFC function and bottom-up emotion processing.

Electro- and magnetoencephalographic (EEG/MEG) studies have revealed that early (∼80–120 ms after stimulus onset) (Keil et al., 2002; Olofsson and Polich, 2007; Feng et al., 2014; Zhu et al., 2015) and mid-latency (∼120–300 ms) (Liddell et al., 2004; Williams et al., 2004; Balconi and Lucchiari, 2005b, 2007; Kiss and Eimer, 2008; Pegna et al., 2008; Kim et al., 2013; Nakajima et al., 2015) event-related potentials and fields (ERPs/ERFs) in response to both subliminal and supraliminal negative material differ from those to positive or neutral material. For example, Liddell et al. (2004) recorded ERPs in response to subliminally (10 ms) and supraliminally (170 ms) presented backward-masked fearful and neutral faces. This study reported main effects for emotion with enhanced frontal (FZ) amplitudes for fearful faces in mid-latency and late components. Further, findings revealed a double dissociation for subliminal and supraliminal fear processing whereby enhanced amplitudes were recorded at mid-latencies over central electrode sites (CZ) for subliminal fearful compared to neutral faces, while similar patterns for supraliminal fear faces were limited to late latencies and frontocentral sites (CZ, FZ). Studies employing source-reconstruction approaches have localized enhanced early and mid-latency responses to negative affective (vs. neutral) visual stimuli in occipital and temporal regions (Junghöfer et al., 2001, 2006; Olofsson et al., 2008; Bayle et al., 2009) and, less consistently, frontal brain regions (e.g., Bayle et al., 2009). These neural responses are thought to reflect mechanisms of “emotional attention,” that is, enhanced automatic orientation to and ongoing preferential encoding of salient stimuli (Halgren and Marinkovic, 1995; Davidson, 2001; Liddell et al., 2004; Williams et al., 2004, 2006). Such mechanism may facilitate a fast feedforward transfer of motivationally relevant information from low- to high-level processing areas (Schupp et al., 2003a,b, 2004a,b; Carretié et al., 2004, 2006; Delplanque et al., 2006; Eimer et al., 2008; Olofsson et al., 2008). Conversely, enhanced late processing (∼300–600 ms) of negative compared to neutral material (Liddell et al., 2004; Kiss and Eimer, 2008; Nakajima et al., 2015) has mainly been found in response to supraliminal stimuli that are available to visual awareness (Liddell et al., 2004; Williams et al., 2004; Balconi and Lucchiari, 2005a,b, 2007; Pegna et al., 2008). Typically, negative–neutral dissociations at late processing stages are supported by distributed posterior and frontal brain networks (Olofsson et al., 2008). Functionally, they have been associated with elaborate processes, including ongoing (perceptual) stimulus evaluation (Leyman et al., 2009; Wessing et al., 2016; for review, see Schupp et al., 2006) and top-down emotion regulation (Kozel and George, 2002; Siegle et al., 2007; Poldrack et al., 2008; Guse et al., 2010; Ochsner et al., 2012; Ironside et al., 2016; Wessing et al., 2016).

The involvement of the prefrontal cortex in the regulation of affective processing is well-documented (Sarter et al., 2001; Bishop et al., 2004; Bressler et al., 2008; Bayle and Taylor, 2010). Despite a large corpus of studies investigating effects of right and left prefrontal rTMS on mood (e.g., Fitzgerald and Daskalakis, 2011) and affective processing (e.g., van Honk et al., 2002; Tupak et al., 2013), the *causal* role of the DLPFC in the modulation neurocognitive mechanisms associated with affective perception of negative, withdrawal-related stimuli has remained largely unclear. To date, only two TMS studies have explored effects of rDLPFC inhibition or excitation on the spatiotemporal dynamics of negative stimulus processing (Zwanzger et al., 2014; Notzon et al., 2017). Zwanzger et al. (2014) recorded magnetoencephalographic ERFs while individuals viewed supraliminal fearful and neutral facial expressions before and after inhibitory repetitive TMS (rTMS) to the rDLPFC or sham stimulation. Results showed that rDLPFC inhibition compared to sham increased early occipito-parietal and mid-latency temporal activations for fearful faces. Likewise, Notzon et al. (2017) reported increased neural activity to fearful faces in right occipital and right temporal regions, after inhibitory compared to excitatory rTMS was applied on the right rDLPFC. Given that such activity has been previously associated with automatic valence-specific feedforward processing of, and emotional attention to both subliminal and supraliminal negative images (Liddell et al., 2004; Williams et al., 2004; Balconi and Lucchiari, 2005a; Balconi, 2006; Balconi and Lucchiari, 2007; Eimer et al., 2008; Kiss and Eimer, 2008; Pegna et al., 2008; Smith, 2012; Kim et al., 2013; Nakajima et al., 2015), the modulatory influence of rDLPFC on early and mid-latency brain signatures of emotion perception might be independent of visual awareness. However, this remains to be tested experimentally, as all previous neurophysiological neurostimulation studies have employed supraliminal stimuli only.

The only behavioral study directly comparing effects of rDLPFC inhibition on subliminal and supraliminal negative processing investigated rTMS effects on top-town control in a modified emotional Stroop task (van Honk et al., 2002). Participants were asked to name the color of masked (subliminal) and unmasked (supraliminal) fearful vs. neutral faces. Findings revealed that inhibitory rDLPFC compared to sham stimulation decreased reaction times for color naming of supraliminal but not subliminal fearful faces. Such reduction of negativity biases following right-hemispheric prefrontal inhibition provides support to theories on hemispheric asymmetries of PFC function (for reviews, see Davidson, 1992a,b; Harmon-Jones et al., 2010; Grimshaw and Carmel, 2014). In this framework, rDLPFC inhibition should reduce right-hemispheric dominance and thereby attenuate negative, withdrawal-related responses. Importantly, van Honk et al. (2002) tentatively suggest that a reduction of negativity biases following rDLPFC inhibition might be limited to stimuli that enter participants’ awareness.

Taken together, current literature indicates that rDLPFC inhibition may on the one hand enhance early bottom-up affect-related brain activation in early and mid-latency ERP/ERF components (Notzon et al., 2017; Zwanzger et al., 2014), which might be independent of stimulus awareness. On the other hand, in presence of visual awareness, rDLPFC inhibition may specifically facilitate elaborate emotion-regulatory behaviors (van Honk et al., 2002). In general, such elaborate mechanisms are reflected in relatively late components (Liddell et al., 2004; Williams et al., 2004; Kiss and Eimer, 2008; Nakajima et al., 2015). However, the spatiotemporal dynamics underpinning awareness-dependent effects of rDLPFC inhibition on affective processing have not yet been revealed.

This study investigated neural and behavioral effects of rDLPFC inhibition on negative scene processing with and without visual awareness. EEG recordings were taken while individuals viewed subliminal and supraliminal negative and neutral natural scenes before (Pre-cTBS) and after (Post-cTBS) inhibitory continuous Theta Burst Stimulation (cTBS, Huang et al., 2005) to the rDLPFC vs. the vertex (Cz, Active Control). Our study followed a 2 × 2 × 2 × 2 mixed factorial design with the between-subject factor Stimulation (rDLPFC inhibition vs. Active Control) and the within-subject factors Session (Pre-cTBS vs. Post-cTBS), Exposure (subliminal vs. supraliminal), and Valence (negative vs. neutral). After the passive viewing task, participants completed several behavioral tasks and self-report mood assessments. Based on these measures we set out to explore the impact of rDLPFC inhibition on (1) affective state, (2) attention mechanisms, (3) valence and arousal ratings, and (4) emotion discrimination in presence and absence of visual awareness.

First, before cTBS application (i.e., Pre-cTBS measurements), we expected enhanced processing of negative compared to neutral images for both subliminal and supraliminal exposure at early (80–120 ms) and mid-latency ERPs (120–300 ms) in occipito-temporal regions (Carretié et al., 2004; Liddell et al., 2004; Junghöfer et al., 2006). As predicted by a feedforward mechanism, such activity was hypothesized to progress toward parietal and frontal regions indexing increasing involvement of higher cortical networks in negative processing. Furthermore, exposure and valence were expected to interact at mid-latency (>120 ms) and/or late intervals (>300 ms) (Liddell et al., 2004; Williams et al., 2004; Kiss and Eimer, 2008; Nakajima et al., 2015). Due to a lack of previous ERP studies employing source localization, clear predictions regarding the underlying neuronal generators were not possible. Based on the above reviewed literature, an involvement of posterior and/or frontal brain regions seemed likely.

Second, and more importantly, we expected differential behavioral and neural effects of rDLPFC inhibition compared to Active Control. Based on van Honk et al. (2002), rDLPFC inhibition was expected to reduce negative processing biases at a behavioral level. On a neuronal level, rDLPFC inhibition was expected to result in increased early (80–120 ms) and mid-latency (120–300 ms) negative-neutral dissociations at occipito-parietal and temporal regions with enhanced activity for negative affective pictures (Zwanzger et al., 2014; Notzon et al., 2017). While these early effects were hypothesized to be independent from visual awareness, interactions of stimulation, exposure, and valence on neural activation differences (Post-cTBS *minus* Pre-cTBS) were predicted for late latencies (>300 ms). In accordance with top-down influences on elaborate processing of negative material (Bishop et al., 2004; Liddell et al., 2004; Kiss and Eimer, 2008; Pegna et al., 2008; Ochsner et al., 2009; Nakajima et al., 2015), an involvement of frontal structures was hypothesized.

## Materials and Methods

### Participants

Forty-eight healthy participants (25 females, 23 males) between 18 and 37 years (*M* = 21.46, *SD* = 4.25) completed the behavioral tasks. EEG was recorded for a subset of 38 participants (22 females, 16 males) between 18 and 35 years (*M* = 21.21, *SD* = 3.81). Participants were recruited at two Hong Kong universities via advertisement and word-of-mouth. Participants received 250 HKD or course credits. In line with the Declaration of Helsinki, the study was approved by the Human Research Ethics Committee for Non-clinical Faculties, The University of Hong Kong, as well as the Human Subjects Ethics Sub-committee of the Hong Kong Polytechnic University. All participants were healthy Chinese Hong Kong locals, fluent in English and with normal or corrected-to-normal vision. Eligibility for receiving rTMS stimulation in accordance with TMS safety guidelines (Grossheinrich et al., 2009; Rossi et al., 2009) was determined using an extended version of the screening standard Transcranial Magnetic Stimulation Safety Questionnaire (TMS-SQ; Rossi et al., 2009). Individuals reporting current or past occurrence of epilepsy, strokes, head- or brain-related medical conditions, loss of consciousness, hearing problems, eye surgery, spinal cord injuries, mental health issues, medication intake, or mental implants were excluded from this study. Further exclusion criteria were pregnancy and family history of epilepsy. Participants were assigned one of two stimulation protocols (rDLPFC vs. Active Control) in a pseudorandom manner so that both groups were matched for gender, age, and professional status. *Post hoc* statistical analyses further confirmed that groups were comparable regarding depression scores [Chinese Version of Beck’s Depression Inventory – II (BDI-II); Beck et al., 1996; Byrne et al., 2004], state and trait anxiety [Chinese Version of State-Trait Anxiety Inventory (STAI); Spielberger et al., 1968; Spielberger, 1983; Shek, 1988, 1993], emotional regulation [Emotion Regulation Questionnaire (ERQ); Gross and John, 2003], and Pre-cTBS mood [Chinese Affect Scale (CAS); Hamid and Cheng, 1996, see **Table 1**].

**Table 1:** Description of the EEG and the Behavioral Samples.

Behavioral sample						
		Sample	rDLPFC inhibition	Active control		
Total *N*		48	24	24		
Gender *N*	Males	23	11	12		
	Females	25	13	12		
Profession *N*	Student	46	23	23		
	Employed	2	1	1		

		***M*(*SD*)**	***M*(*SD*)**	***M*(*SD*)**	***F*(1,47)**	***p***

Age (years)		21.45 (4.25)	21.08 (3.76)	21.83 (4.74)	0.368	0.547
BDI		0.42 (0.31)	0.36 (0.22)	0.49 (0.38)	1.986	0.166
STAI (state)		36.94 (9.18)	36.13 (9.40)	37.75 (9.09)	0.371	0.546
STAI (trait)		45.13 (10.39)	44.92 (10.24)	45.33 (10.75)	0.019	0.891
ERQ (reappraisal)		5.33 (0.78)	5.53 (0.74)	5.13 (0.79)	3.199	0.080
ERQ (suppression)		4.51 (1.24)	4.65 (1.09)	4.38 (1.38)	0.569	0.454
CAS (pos)		2.24 (0.84)	2.31 (0.81)	2.16 (0.88)	0.400	0.530
CAS (neg)		1.09 (0.95)	1.10 (0.94)	1.08 (0.98)	0.006	0.940
**EEG sample (subgroup of behavioral sample)**						
		**Sample**	**rDLPFC inhibition**	**Active control**		

Total *N*		38	19	19		
Gender *N*	Males	16	8	8		
	Females	22	11	11		
Profession *N*	Student	36	18	18		
	Employed	2	1	1		

		***M*(*SD*)**	***M*(*SD*)**	***M*(*SD*)**	***F*(1,36)**	***p***

Age (years)		21.21 (3.81)	20.42 (1.89)	22.00 (5.00)	1.657	0.206
BDI		0.39 (0.32)	0.35 (0.17)	0.43 (0.41)	0.771	0.386
STAI (state)		1.91 (0.43)	1.99 (0.30)	1.93 (0.54)	0.057	0.813
STAI (trait)		2.22 (0.48)	2.15 (0.34)	2.30 (0.58)	0.913	0.346
ERQ (reappraisal)		5.35 (0.81)	5.52 (0.68)	5.19 (0.91)	1.555	0.221
ERQ (suppression)		4.51 (1.12)	4.53 (0.83)	4.47 (1.36)	0.032	0.859
CAS (pos)		2.20 (0.80)	2.27 (0.19)	2.13 (0.84)	0.290	0.593
CAS (neg)		1.04 (0.91)	1.08 (0.83)	0.99 (0.99)	0.102	0.752

*BDI, Becks Depression Inventory; STAI, State-Trait-Anxiety-Inventory; ERQ, Emotion Regulation Questionnaire; reapp, reappraisal; sup, suppression; CAS, Chinese Affect Scale; pos, positive; neg, negative; *M*, mean, *SD*, standard deviation, rTMS, repetitive transcranial magnetic stimulation.*

### Procedure

**FIGURE 1:**
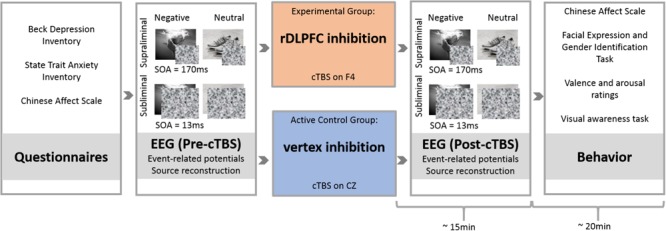
Experimental paradigm. After completing questionnaires, participants were assigned to either the experimental or the Active Control group. In the experimental group (rDLPFC inhibition group), rDLPFC excitability was reduced by administration of inhibitory continuous Theta Burst Stimulation (cTBS) to electrode F4. The Active Control group received cTBS on CZ (vertex). Before and after cTBS-application (Pre-cTBS vs. Post-cTBS), participants viewed four blocks of backward-masked supraliminal (SOA = 170 ms) and subliminal (SOA = 13 ms) negative (e.g., shark) and neutral (e.g., shoes) scenes. Thereby, each of the overall eight blocks contained a different set of 50 images to avoid the emergence of familiarity effects or habituation through repetition of identical images across blocks. Within each block, each image was presented three times. The assignment of images to blocks was counterbalanced across subjects. Following EEG measurements, participants completed behavioral tasks in order to evaluate effects of cTBS stimulation on self-reported mood (CAS), on attention engagement to and disengagement from emotional stimuli (Facial Expression and Gender Identification Task), on valence and arousal ratings of images, and on discrimination of negative versus neutral images under supra- and subliminal viewing conditions (visual awareness task). All post-cTBS measurements were completed within 35 min after stimulation.

Individuals filled in the TMS-SQ prior to the experimental session and only those eligible for safe rTMS administration were invited to attend. Individual sessions were conducted in a quiet room after participants had learned about the procedures and equipment and given written informed consent. Participants first completed BDI-II, STAI, ERQ, and CAS. Then, EEG cap and corresponding ocular electrodes were adjusted on the head of the participant and Pre-cTBS EEG activity was recorded while participants were exposed to the passive viewing presentation. No response was required during the presentation. In line with prior research (Liddell et al., 2004), participants were instructed to concentrate on the images and told they may need to answer questions about these images afterward. Subsequently, offline cTBS was administered on rDLPFC or vertex, the Active Control region, before participants viewed the Post-cTBS presentation accompanied by EEG recordings. Upon completion, participants again filled the CAS and then performed five behavioral tasks in the following order: facial expression identification, facial gender identification (Nitsche et al., 2012; Zwanzger et al., 2014; Conson et al., 2015), valence and arousal evaluation of images (Feeser et al., 2014; Zwanzger et al., 2014; Balconi and Cobelli, 2015; Era et al., 2015), and detection and discrimination visual awareness tasks (Liddell et al., 2004; Williams et al., 2004). After conclusion of the experiment, participants were thoroughly debriefed and received contact details of the experimenter, supervisor, ethics committee, and university counseling services. The entire session took approximately 110 min (**Figure 1**).

### cTBS Protocol

To inhibit the rDLPFC, a continuous Theta-Burst Stimulation protocol (cTBS; Huang et al., 2005) was applied over the right F4 electrode using a Magstim stimulator (Magstim, Morrisville, NC, United States^1^) with a 70-mm figure-eight shaped double coil (Grossheinrich et al., 2009; Cho et al., 2010; Zwanzger et al., 2014). Explicitly, 200 bursts of three pulses at 50 Hz each were administered with a frequency of 5 Hz which resulted in a total of 600 pulses, i.e., train of three pulses was repeated every 200 ms for approximately 42 s (Cho et al., 2010; Oberman et al., 2011). Such brief cTBS has been associated with temporarily reduced cortical excitability for approximately 45 times stimulation length, i.e., around 30 min for 42 s stimulation (Klimesch, 1996; Oberman et al., 2011). The Active Control stimulation was administered on the vertex at electrode Cz, which is a region often targeted in control conditions (Kwan et al., 2007; Balconi and Ferrari, 2013) and which was shown to play a minor role in emotion processing, as revealed by a recent meta-analysis of 157 fMRI studies on emotional face and emotional scene processing (Sabatinelli et al., 2011). By choosing an active-controlled design, we intended to match the sensory experience of experimental and control group as closely as possible. Considering mixed empirical evidence for the assumption that individual motor or phosphene thresholds capture site-nonspecific factors of cortical excitability that will generalize to other brain areas (Boroojerdi et al., 2002; Gerwig et al., 2003; Antal et al., 2004; Stokes et al., 2012), we stimulated all participants with a fix intensity of 50% maximal stimulator output. All safety requirements regarding frequency, length, and stimulation intensity were adhered to (Wassermann, 1998; Machii et al., 2006; Rossi et al., 2009; Oberman et al., 2011). No side effects or discomfort were reported.

### Stimuli

For the passive viewing paradigm, we selected 200 negative and 200 neutral color images from well-established databases, including the International Affective Picture System (IAPS) (Lang et al., 1999, 2005; Gong and Wang, 2016), the Chinese Affective Picture Systems (CAPS) (Lu et al., 2005), and the Geneva Affective Picture Database (GAPED) (Dan-Glauser and Scherer, 2011). Pre-existing valence and arousal ratings for all images (Lang et al., 1999, 2005; Lu et al., 2005; Dan-Glauser and Scherer, 2011) were collapsed onto a nine-point scale in accordance with existing IAPS ratings. Valence and arousal ratings ranged from low to high, indicating negative-to-positive valence and low-to-high arousal, respectively. *T*-tests confirmed that negative images were rated as significantly lower in valence (*M* = 2.44, *SD* = 0.93) and higher in arousal (*M* = 6.05, *SD* = 0.93) than neutral images [valence: *M* = 5.37, *SD* = 0.73; arousal: *M* = 3.29, *SD* = 1.40; *t*(398) = -34.982, *p* < 0.001, *t*(398) = 23.19, *p* < 0.001, respectively].

All images were transformed into grayscale and luminance-matched via the SHINE toolbox (Willenbockel et al., 2010a,b) on Matlab 2008a (Mathworks, Natick, MA, United States^2^). This toolbox computes an average luminance histogram from the histograms of all images and matches all images to this reference [Negative: *M* = 104.25, *SD* = 5.70; Neutral: *M* = 104.32, *SD* = 5.67; *t*(398) = -0.114, *p* = 0.909]. Scrambled masks were made from each image, rendering its contents unrecognizable. The Matlab-based script calculated total pixels per image, and randomly changed the position of each pixel while keeping image width and height constant. Each negative and neutral image was followed by its corresponding scrambled mask.

### Experimental Task: Passive Viewing Paradigm

For the EEG recordings, participants sat in a quiet, dimly lit room. Visual images were presented centrally on a 13in SVGA monitor with a 1920 × 1080 resolution (refresh rate: 75 Hz), which was situated 60 cm from participants’ eyes. Horizontal images were 195 × 260 px in size with a visual angle of 4 × 6° and vertical pictures were 142 × 195 px in size with a visual angle of 4 × 4.65°. As in previous EEG studies on subliminal emotion perception, backward-masked stimuli were presented in a block-design (Liddell et al., 2004; Williams et al., 2004) against a black background. Before and after cTBS application (Pre-cTBS vs. Post-cTBS), participants viewed four blocks of subliminal-neutral, supraliminal-neutral, subliminal-negative, and supraliminal-negative images. Thereby, each of the overall eight blocks contained a different set of 50 images to avoid the emergence of familiarity effects or habituation through frequent repetition of identical images. Stimuli were presented by means of E-Prime 2.0 Professional Software (Psychology Software Tools, Inc., Sharpsburg, PA, United States^3^). Within each block, 50 negative or neutral images were presented in random order and repeated three times. This resulted in 150 images per block and 600 images per presentation. Participants could choose to take a short break between blocks. To avoid confounding effects of order or stimulus sets, the order of blocks and the assignment of stimuli to experimental blocks were counterbalanced across participants using the Latin Square system.

Subliminal images were presented for 13 ms, supraliminal images for 170 ms. All images were preceded by a jittered inter-stimulus fixation cross (700–1000 ms) and succeeded by a scrambled mask (170 ms). Visual angles and exposure times were in accordance with those shown in previous subliminal research to ensure image content remains unreportable above chance (Liddell et al., 2004; Hsu et al., 2008; Japee et al., 2009; Ibáñez et al., 2011; Lee et al., 2011; Pegna et al., 2011; Li and Lu, 2014; Nakajima et al., 2015).

### EEG Recording and Analysis

Electroencephalographic signals were recorded in a sound-attenuated chamber with low lighting and electromagnetic shield, using a 64-channel TMS-compatible EEG cap (i.e., with non-magnetic electrodes and cables and flat electrodes to minimize TMS-coil to scalp distance; Easycap GmbH, Germany, Asian head shape) according to the International 10–20 system (Blom and Anneveldt, 1982). During recording, FCz was used as a reference point. Horizontal and vertical eye movement potentials were recorded via four ocular electrodes placed 1 cm from the outer canthus of each eye and 1 cm above and below the left eye. Following prior research (Smith, 2012; Hintze et al., 2014), all electronic impedances were kept at less than 10 kΩ and data were recorded continuously at a sampling rate of 1000 Hz. Signals were amplified via SynAmp2 and an online anti-aliasing low-pass filter was applied (Neuroscan Compumedics Ltd., Australia ^4^).

Electroencephalographic data preprocessing was conducted via Curry 7 (Neuroscan Compumedics Ltd., Australia). Offline, the raw data were resampled to 250 Hz, and re-referenced to average reference. Data were filtered with a digital 0.1 Hz high-pass and a 35 Hz low-pass filter. ERP epochs from 200 ms pre-stimulus to 600 ms post-stimulus were computed separately for the four within-subject conditions (supraliminal-negative, supraliminal-neutral, subliminal-negative, subliminal-neutral) for rDLPFC inhibition and Active Control groups. The pre-stimulus interval from -200 ms to stimulus onset was used for baseline correction. Artifact rejection and eye blink correction were conducted and trials with amplitudes ±70 μV were rejected. This procedure led to rejection of an average of 14.3% of trials, equally distributed across all conditions [session × exposure × valence × stimulation; *F*(1,36) = 0.255, *p* = 0.617], which was deemed acceptable given prior research (Smith, 2012; Nomi et al., 2013; Hintze et al., 2014).

Separately for our experimental conditions, we then estimated the current sources for the averaged epochs using the L2-Minimum-Norm-Estimates approach (L2-MNE) (Hämäläinen and Ilmoniemi, 1994) and a spherical head model with evenly distributed 3 (radial, azimuthal, and polar direction) × 197 dipoles (see Hintze et al., 2014). The L2-MNE technique does not make prior assumptions regarding location of number of sources, but instead extracts generators based on the distribution of electric potential across the head sphere (Hämäläinen and Ilmoniemi, 1994; Hauk, 2004). Across all participants and conditions, the Tikhonov regularization parameter *k* was set at 0.1.

Two main analyses were conducted. First, to replicate previous affective processing literature, a 2 × 2 factorial repeated measures ANOVA was conducted on Pre-cTBS data with the factors exposure (supraliminal vs. subliminal) and valence (negative vs. neutral). Second, to investigate the effects of rDLPFC inhibition on subliminal and supraliminal emotion processing, difference scores for neural responses were calculated by subtracting Pre-cTBS from Post-cTBS ERPs. These scores were submitted to a 2 × 2 × 2 mixed ANOVA with the factors exposure (supraliminal vs. subliminal), valence (negative vs. neutral), and stimulation (rDLPFC inhibition vs. Active Control).

A non-parametric statistical testing procedure that included correction for multiple comparisons (Maris and Oostenveld, 2007), similar to cluster-based permutation approaches used in hemodynamic imaging, was applied to reveal effects of interest. As part of this procedure, *F*-values of spatially neighboring (minimally five neighboring dipoles) and temporally consecutive (minimally five consecutive time points) test dipoles below a critical alpha level of *p* = 0.05 (sensor-level criterion) were summed up to so-called cluster masses. Based on prior research, the current study separately investigated time windows consistently reported to show affect-specific neural potentials during both supraliminal and subliminal negative facial expression processing. Correspondingly, the time windows of interest were defined as early from 80 to 120 ms (Carretié et al., 2004; Jiang et al., 2009; Keuper et al., 2013; Nomi et al., 2013; Li and Lu, 2014), mid-latency from 120 to 300 ms (Junghöfer et al., 2001; Carretié et al., 2004; Jiang et al., 2009; Kim et al., 2013) and late from 300 to 600 ms (Schupp et al., 2000, 2006; Olofsson et al., 2008; Pegna et al., 2011; Kim et al., 2013). To avoid latency biases toward late processes with much stronger and more sustained neural activations, cluster masses of relevant effects were calculated separately for the early (80–120 ms), mid-latency (120–300 ms), and late (300–600 ms) post-onset time intervals. They were tested against a random cluster-based permutation alpha level of *p* = 0.05, which was established via Monte Carlo simulations of identical analyses based on 1000 permuted drawings of experimental data sets (i.e., the *F* distributions for each time interval were built up by the 1000 clusters with the biggest masses within each time interval). Thus, only cluster masses exceeding an alpha level of *p* = 0.05 within each time interval were considered (cluster-level criterion). All significant spatiotemporal clusters with a minimum interval length of 10 ms and three neighboring source dipoles (Zwanzger et al., 2014) were further delineated in *post hoc* Bonferroni corrected paired and independent *t*-tests.

### Behavioral Tasks and Analyses

Participants completed behavioral tasks following the Post-cTBS EEG-measurement in order to evaluate behavioral effects of stimulation on self-reported mood (CAS, Hamid and Cheng, 1996), on attention engagement to and disengagement from facial stimuli with fearful and neutral expressions (Facial Expression and Gender Identification Task, Zwanzger et al., 2014), on valence and arousal ratings of negative and neutral images, and on discrimination of negative and neutral images under supra- and subliminal viewing conditions (visual awareness task; Liddell et al., 2004; Williams et al., 2004). All tasks were conducted within a time-window of 35 min after cTBS application.

#### Chinese Affect Scale (CAS)

The CAS is a self-report scale measuring negative and positive affective mood states that has been specifically created for the Chinese population. It is reported to have high internal (α > 0.89) and moderate re-test reliabilities (*r* = 0.43–0.47) (Hamid and Cheng, 1996). Importantly, it has been shown to adequately capture minor changes in momentary mood state (Hamid and Cheng, 1996). Using independent-sample *t*-tests, changes (Post-cTBS *minus* Pre-cTBS) in positive and negative affect ratings were compared between the rDLPFC inhibition group and the Active Control group.

#### Facial Expression and Gender Identification Task

In the facial expression and gender identification task, participants were asked to either indicate the facial expression or the gender of 80 supraliminal gray-scale face images (40 males, 40 females) showing fearful (i.e., negative, 50%) and neutral (50%) facial expressions. The paradigm has been validated in a prior study exploring the impact of rTMS on affective processing (for details, please see Zwanzger et al., 2014). Reaction times and accuracy were recorded for both tasks and submitted to four separate 2 × 2-mixed ANOVAs with the within-subject factor valence (negative vs. neutral), the between-subjects factor stimulation (rDLPFC inhibition vs. Active Control). The Facial Expression and Gender Identification tasks were administered with the goal to identify between-group differences in negative biases due to enhanced attention orienting to and/or reduced attention disengagement from negative compared to neutral stimuli following rDLPFC inhibition. Preferential emotional attention orienting is typically reflected in the faster detection of negative compared to neutral stimuli. By contrast, tasks requiring disengagement of emotional attention toward non-emotional characteristics of stimuli (e.g., gender identification task) typically reveal slower reaction times for negative stimuli.

#### Valence and Arousal Ratings

In the valence and arousal ratings, participants were exposed to 25 neutral and 25 negative images (195 × 260 px, visual angle 4 × 6°) that had been randomly chosen from the 400 images of the passive viewing paradigm. These images were presented against a white background and remained on the screen until participants had given their responses. For each image, participants rated valence and arousal on two separate computerized Visual Analogue Scales (VAS), which ranged from extremely negative to extremely positive (0–100), and not aroused to extremely aroused (0–100), respectively. Valence and arousal ratings were submitted to two separate 2 × 2-mixed ANOVAs with the within-subject factor valence (negative vs. neutral) and the between-subjects factor stimulation (rDLPFC inhibition vs. Active Control).

#### Visual Awareness Task

In the visual awareness task (Liddell et al., 2004; Williams et al., 2004), we presented 96 negative and neutral images, randomly chosen from the passive viewing paradigm, in a subliminal or supraliminal block using E-prime 2.0 Professional. Images were preceded by a fixation cross of 500 ms, but otherwise followed the same parameters as the passive viewing paradigm. Participants were required to make a forced-choice decision after each image, indicating whether image contents were negative or neutral (discrimination task). This task was administered to confirm that subliminal images were not reportable above chance and to assess effects of rDLPFC inhibition on discrimination abilities. D-prime scores close to 0 (*d′* = 0) and greater than 1 (*d′* > 1), respectively, confirm that images did or did not remain subliminal below visual awareness (Green and Swets, 1966; Eimer et al., 2008; Pegna et al., 2011; Smith, 2012; Zhang et al., 2012). In order to test for effects of stimulation on visual awareness, *d*’ scores of the subliminal and the supraliminal block were submitted to two separate 2 × 2-way ANOVAs with the within-subject factors valence (negative vs. neutral) and the between-subjects factor stimulation (rDLPFC inhibition vs. Active Control).

## Results

### Valence and Exposure Effects in Neurophysiological Pre-cTBS Measures

**FIGURE 2:**
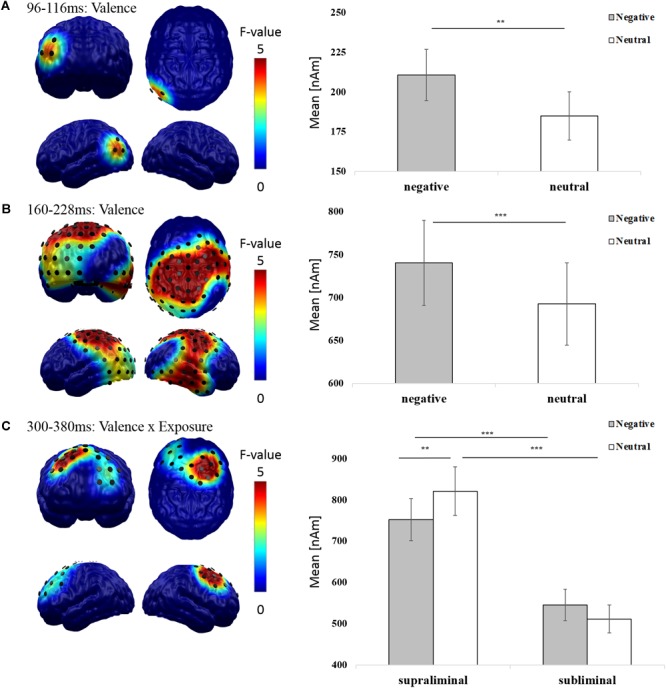
Valence and exposure effects in neurophysiological Pre-cTBS data. The distribution of mean *F*-values for the **(A)** early (96–116 ms) and **(B)** mid-latency (160–228 ms) main effect of valence and the **(C)** late (300–380 ms) interaction of valence and exposure are displayed for all significant spatiotemporal clusters and masked at a significance level of *p* < 0.05 (sensor-criterion). Dipoles included in the spatiotemporal cluster (*p* < 0.05, corrected) are superimposed in black color. The bar plot depicts the regional mean neural activity in the respective spatiotemporal clusters in response to negative (gray) and neutral (white) images. Error bars indicate the standard errors of the means. Significant results of *post hoc t*-tests (Bonferroni corrected) are indicated (^∗^*p* < 0.05, ^∗∗^*p* < 0.01, ^∗∗∗^*p* < 0.001).

In order to replicate main effects of emotion and interactions of emotion and exposure, we separately investigated early (80–120 ms), mid-latency (120–300 ms), and late epochs (300–600 ms; e.g., Junghöfer et al., 2001; Schupp et al., 2006; Keuper et al., 2013) using 2 × 2 factorial repeated measures ANOVAs with the factors exposure (supraliminal vs. subliminal) and valence (negative vs. neutral). These revealed two significant clusters for the main effect of valence with greater amplitudes for negative than neutral images. First, in an early (96–116 ms) time window, effects were located in left occipital regions [*F*(1,37) = 7.963; *p* = 0.008; **Figure 2A**]. Second, at mid.latencies (160–228 ms) effects were found in left occipito-parietal and right temporo-parietal areas [*F*(1,37) = 15.220; *p* < 0.001; **Figure 2B**]. No late main effects were found for valence.

We further found a significant interaction between exposure and valence at late latencies (300–380 ms) in a frontal cluster with pronounced right hemisphere activation [*F*(1,37) = 12.676, *p* = 0.001; **Figure 2C**]. *Post hoc t*-tests demonstrated that this interaction was driven by increased amplitudes to subliminal negative compared to neutral images [*t*(37) = 3.149, *p* = 0.003]. Supraliminal compared to subliminal images elicited enhanced overall amplitudes in both valence conditions [negative: *t*(37) = 8.320, *p* < 0.001; neutral: *t*(37) = 10.434, *p* < 0.001]. No significant difference was found between amplitudes elicited by supraliminal negative compared to neutral images [*t*(37) = -1.300, *p* = 0.202].

### Effects of rDLPFC Inhibition on Behavioural Measures

#### Chinese Affect Scale: No Effects of rDLPFC Inhibition on Self-Reported Mood

Independent-sample *t*-tests revealed no effects of stimulation on self-reported mood [negative affect: *t*(46) = 0.768, *p* = 0.447; positive affect: *t*(46) = -0.514, *p* = 0.610]. These findings suggest that rDLPFC inhibition did not affect the immediate affective state.

#### Facial Expression and Gender Identification Task: Effects of rDLPFC Inhibition on Emotional Attention Engagement and Disengagement

**FIGURE 3:**
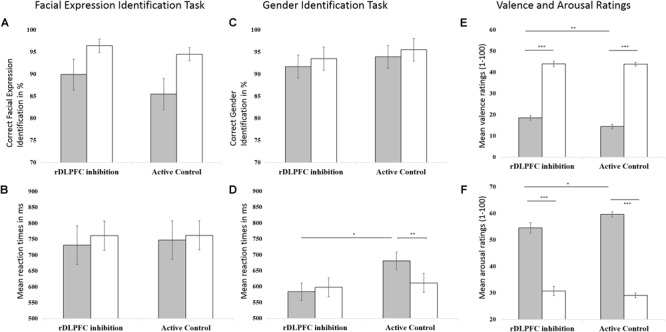
Effects of stimulation (rDLPFC inhibition vs. Active Control) and valence (negative vs. neutral) on behavioral responses. Left column: **(A)** Mean response accuracies and **(B)** reaction times to negative (here: fearful; gray) and neutral (white) facial expressions in the Facial Expression Identification Task. Middle column: **(C)** Mean response accuracies and **(D)** reaction times to negative (here: fearful; gray) and neutral (white) facial expressions in the Gender Identification Task. Right Column: **(E)** Mean valence ratings and **(F)** arousal ratings in response to negative (gray) and neutral (white) images. Higher scores indicate more positive and higher arousal ratings, respectively. Error bars indicate the standard errors of the means. For significant interactions of valence and stimulation, results of *post hoc t*-tests (Bonferroni corrected) are indicated (^∗^*p* < 0.05, ^∗∗^*p* < 0.01, ^∗∗∗^*p* < 0.001).

To explore effects of stimulation on valence identification, we investigated response accuracies and reaction times of the Facial Expression Identification task using a 2 × 2-mixed ANOVAs with within-subjects factor valence and between-subjects factor stimulation (**Figures 3A,B**). Data revealed a significant main effect of valence on response accuracy [*F*(1,46) = 11.559, *p* = 0.001] with fewer correct responses for fearful (*M* = 0.88, *SD* = 0.17) compared to neutral faces (*M* = 0.96, *SD* = 0.08). There were neither a main effect of stimulation [*F*(1,46) = 0.658, *p* = 0.421] nor an interaction of valence and stimulation [*F*(1,46) = 0.457, *p* = 0.503] on response accuracy. Reaction times for facial expression identification were not affected by valence [*F*(1,46) = 0.515, *p* = 0.477], stimulation [*F*(1,46) = 0.014, *p* = 0.907], or their interaction [*F*(1,46) = 0.051, *p* = 0.822].

To explore stimulation effects on attentional disengagement from negative material, we investigated response accuracy and reaction times in the facial gender identification task. As rDLPFC inhibition was shown to facilitate attention disengagement (van Honk et al., 2002), we expected relatively higher response accuracies and/or faster reaction times specifically for the gender identification of negative faces following rDLPFC inhibition compared to Active Control. We found a significant main effect of valence on response accuracy [*F*(1,46) = 11.806, *p* = 0.001] with fewer correct responses for fearful (*M* = 0.93, *SD* = 0.12) compared to neutral faces (*M* = 0.95, *SD* = 0.12, **Figure 3C**). These findings are in line with research demonstrating emotional interference effects, which suggest that it is more difficult to disengage from emotional compared to neutral material, as emotional materials capture attention. Response accuracies were not affected by stimulation [*F*(1,46) = 0.338, *p* = 0.564] nor by the interaction between valence and stimulation [*F*(1,46) = 0.041, *p* = 0.841]. However, we found a significant interaction between stimulation and valence for reaction time [*F*(1,46) = 4.073, *p* = 0.049, **Figure 3D**]. While participants in the Active Control condition showed longer reaction times to fearful (*M* = 681.16 ms, *SD* = 155.10 ms) than to neutral facial expressions [*M* = 611.19 ms, *SD* = 116.22 ms, interference_Neg-Neu_: *M* = 69 ms, *SD* = 107.27 ms; *t*(23) = 3.194, *p* = 0.004], this effect was absent in the rDLPFC inhibition group [interference_Neg-Neu_: *M* = -14.04 ms, *SD* = 173.41 ms, *t*(23) = -0.397, *p* = 0.695]. Compared to the Active Control group, the rDLPFC inhibition group showed significantly shorter reaction times to fearful [*M* = 583.94 ms, *SD* = 108.68 ms; *t*(46) = 2.515, *p* = 0.015] and similar reaction times to neutral [*t*(46) = 0.315, *p* = 0.754] facial expressions. No significant main effects of stimulation [*F*(1,46) = 2.558, *p* = 0.117] or valence [*F*(1,46) = 1.805, *p* = 0.186] were found for reaction time. These findings indicate that rDLPFC inhibition by cTBS speeded up successful attentional disengagement from negative material, while interference effects in response accuracies remained unaffected.

#### Valence and Arousal Ratings: Effects of rDLPFC Inhibition on Image Evaluations

To investigate effects of stimulation on explicit image evaluations, two 2 × 2-mixed ANOVAs with within-subjects factor valence and between-subjects factor stimulation were conducted for valence and arousal ratings of negative and neutral images.

For valence ratings (**Figure 3E**), there were significant main effects of valence [*F*(1,46) = 671.52, *p* < 0.001] and stimulation [*F*(1,46) = 3.873, *p* = 0.05] as well as a significant interaction between valence and stimulation [*F*(1,46) = 3.800, *p* = 0.05]. Bonferroni-corrected *t*-tests showed that for both rDLPFC inhibition and Active Control group, negative images received lower valence ratings than neutral images [rDLPFC inhibition: Negative: *M* = 18.54, *SD* = 5.39, Neutral: *M* = 43.94, *SD* = 5.97, *t*(46) = -14.599, *p* < 0.001; Active Control: Negative: *M* = 14.35, *SD* = 5.15, Neutral: *M* = 43.88, *SD* = 4.34, *t*(46) = -24.385, *p* < 0.001]. Crucially, for negative images, individuals in the rDLPFC inhibition group gave significantly less negative (i.e., higher) valence ratings (*M* = 18.54, *SD* = 5.39) than those in the Active Control group [*M* = 14.35, *SD* = 5.15, *t*(46) = -2.756, *p* = 0.008]. No significant differences for neutral images were observed [*t*(46) = -0.041, *p* = 0.968].

For arousal ratings (**Figure 3F**), a significant main effect of valence [*F*(1,46) = 264.463, *p* < 0.001] and a significant interaction between valence and stimulation [*F*(1,46) = 4.064, *p* = 0.05] were observed. The main effect of stimulation was not significant [*F*(1,46) = 1.829, *p* = 0.183]. Not surprisingly, Bonferroni-corrected *t*-tests revealed that for both rDLPFC inhibition and Active Control conditions, negative images received higher arousal ratings than neutral images [rDLPFC inhibition: Negative: *M* = 54.49, *SD* = 9.68, Neutral: *M* = 30.72, *SD* = 8.74, *t*(23) = -7.831, *p* < 0.001; Active Control: Negative: *M* = 59.60, *SD* = 4.70, Neutral: *M* = 29.04, *SD* = 4.55, *t*(23) = 20.951, *p* < 0.001]. Importantly, for negative images, arousal ratings were significantly lower in the rDLPFC inhibition compared to the Active Control condition [*t*(46) = 2.324, *p* = 0.025] while no significant difference was detected for neutral images [*t*(46) = -0.837, *p* = 0.407].

#### Visual Awareness Task: No Effects of rDLPFC Inhibition on Discrimination of Subliminal and Supraliminal Images

The visual awareness task at the end of the experimental session showed that participants were unable to report the emotional content of subliminally presented images above chance [*t*(47) = 0.116, *p* = 0.908; *d′*: *M* = 0.22, *SD* = 0.51]. In contrast, as expected, supraliminal stimuli could be discriminated above chance [*t*(47) = 11.304, *p* < 0.001, *d′*: *M* = 1.89, *SD* = 0.98]. Discrimination of both subliminal and supraliminal stimuli remained unaffected by the stimulation [*F*(1,47) < 1].

### Effects of rDLPFC Inhibition on Neurophysiological Measures

#### Awareness-Independent Effects of rDLPFC Inhibition: Automatic Valence Processing

**FIGURE 4:**
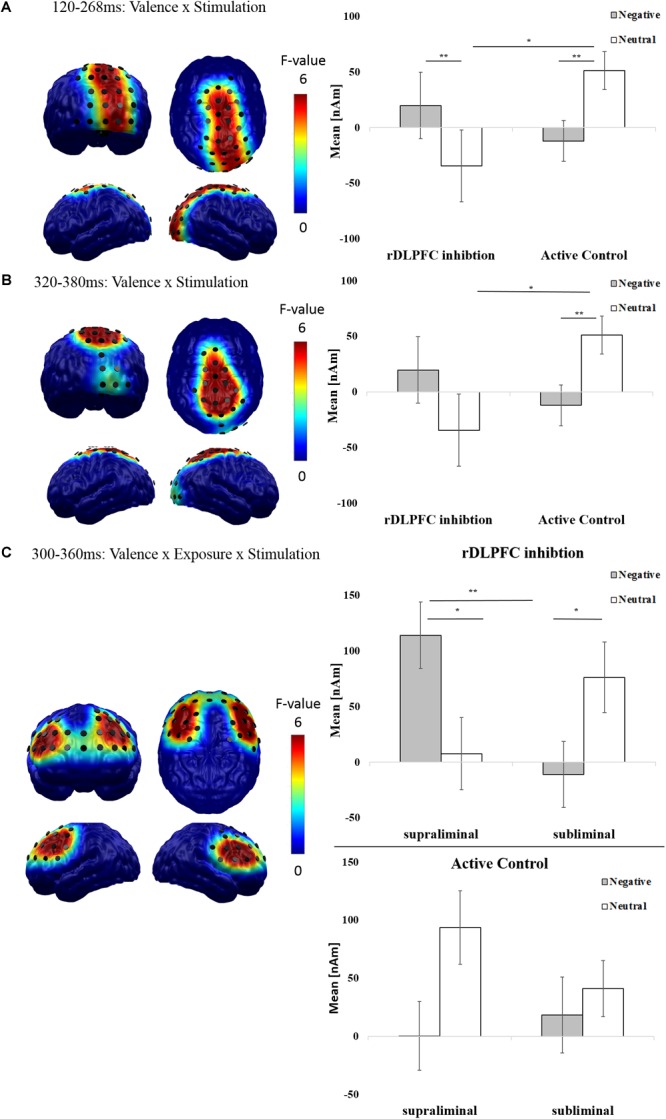
Effects of rDLPFC inhibition on neurophysiological measures of affective processing: The spatial distribution of mean *F*-values for the **(A)** mid-latency (120–268 ms) and **(B)** late (320–380 ms) interaction of valence and stimulation, and **(C)** for the late (300–360 ms) interaction of valence, stimulation and exposure are displayed for all significant spatiotemporal clusters and masked at a significance level of *p* < 0.05 (sensor-criterion). Dipoles included in the spatiotemporal cluster (*p* < 0.05, corrected) are superimposed in black color. The bar plot depicts the regional mean neural difference activity (Post-cTBS *minus* Pre-cTBS, in nAm) in the respective spatiotemporal clusters in response to negative (gray) and neutral (white) scenes. Error bars indicate the standard errors of the means. Results of *post hoc t*-tests for significant interactions (Bonferroni corrected) are indicated (^∗^*p* < 0.05, ^∗∗^*p* < 0.01, ^∗∗∗^*p* < 0.001).

In a first step, to investigate stimulation–valence interactions that are independent of visual awareness, we calculated spatiotemporal clusters for the interaction of valence and stimulation. We found a significant cluster for the interaction between stimulation and valence within the mid-latency time window [120–268 ms; *F*(1,36) = 22.299, *p* < 0.001; **Figure 4A**] in an occipito-parietal brain area and within the late time window [320–380 ms; *F*(1,36) = 9.759, *p* = 0.004] in fronto-parietal areas (**Figure 4B**). *Post hoc* analyses for the mid-latency cluster revealed that, following rDLPFC inhibition, negative compared to neutral images elicited an increase in amplitude from Pre-cTBS to Post-cTBS [*F*(1,36) = 10.730, *p* = 0.004]. Conversely, in the Active Control condition, negative compared to neutral images elicited a relative decrease in amplitude from Pre-cTBS to Post-cTBS [*F*(1,18) = 11.601, *p* = 0.003]. In the late cluster, we observed effects in similar directions: While enhanced negative compared to neutral brain activity following rDLPFC inhibition failed to reach significance, negative images elicited reduced amplitudes from Pre-cTBS to Post-cTBS in the Active Control condition [*F*(1,36) = 13.595, *p* = 0.002].

As mid-latency effects started at the boundary between the early and mid-latency interval of interest, we conducted a follow-up analyses correcting for a merged time window between 80 and 300 ms. This analysis revealed that the interactive effect of valence and stimulation began to show significance at 112 ms. This early onset of the valence × stimulation interaction, which remains affected by the factor exposure, is in line with the claim that rDLPFC modulation enhances rather automatic neural processes reflecting motivated attention (e.g., Zwanzger et al., 2014).

#### Awareness-Dependent Effects of rDLPFC Inhibition: Elaborate Valence Processing

To reveal spatiotemporal clusters in which valence × stimulation interactions are modulated by visual awareness, we calculated cluster masses for the interaction between stimulation, valence, and exposure. While analyses in the early and mid-latency interval yielded no significant clusters, we found a significant cluster in the late time window (300–360 ms) at frontal sites [*F*(1,37) = 11.527, *p* = 0.002; **Figure 4C**]. As this late effect started at the boundary between the mid-latency and the late interval, we conducted follow-up tests for a merged time window (120–600 ms), which showed that the late activation began to show significance at 292 ms.

*Post hoc* 2 × 2 within-subjects ANOVAs with the factors valence and exposure for this late frontal spatio-temporal cluster were conducted separately for rDLPFC inhibition and Active Control groups. A significant interaction between exposure and valence was found for the rDLPFC inhibition condition [*F*(1,18) = 28.474, *p* < 0.001]. Bonferroni-corrected paired *t*-tests demonstrated that this interaction was driven by increased amplitudes for supraliminal negative images compared to neutral [*t*(18) = 2.379, *p* = 0.029] and compared to subliminal negative images [*t*(18) = 3.569, *p* = 0.002]. Crucially, a reverse pattern was noted for subliminal material, whereby subliminal neutral compared to negative images [*t*(18) = -2.474, *p* = 0.024] and supraliminal neutral images [trend toward significance; *t*(18) = -1.933, *p* = 0.062] elicited increased activity. By contrast a significant main effect of valence was found in the Active Control condition [*F*(1,18) = 6.007, *p* = 0.025] with increased amplitudes from Pre-cTBS to Post-cTBS for neutral compared to negative images.

These findings suggest that at later processing stages, rDLPFC inhibition results in enhanced affect-specific activation of dorsolateral brain structures only if negative images enter visual awareness. Based on the topography and the late onset of this awareness-dependent effect, it is plausible that it is linked to elaborate mechanisms such as emotion-regulation or attention disengagement (van Honk et al., 2002; Liddell et al., 2004).

## Discussion

The rDLPFC plays a key role in the regulation of emotional processing (Ochsner et al., 2012). However, its specific role in the regulation of neurocognitive processes that underpin the interplay of affective perception and visual awareness is largely unknown. To address this research gap, the current study experimentally induced rDLPFC inhibition using inhibitory cTBS. In an active-controlled mixed factorial design, we studied the effects of this stimulation on subsequent behavioral and electroencephalographic responses to subliminally and supraliminally presented negative versus neutral scenes. We applied minimum-norm approaches to estimate the corresponding neuronal sources. In the following, we will first discuss findings of our EEG Pre-cTBS measurement and thereby consider how visual awareness affects the spatiotemporal dynamics of emotion processing under passive viewing conditions. Second, the key question of this study will be addressed: In which way does rDLPFC inhibition influence behavioral and neurophysiological correlates of emotional responses to supraliminal and subliminal visual stimuli? The discussion offers potential mechanisms and highlights areas of future investigation.

### Valence and Exposure Effects in Neurophysiological Pre-cTBS Measures

In our neurophysiological Pre-cTBS measures, we could replicate enhanced brain activation in response to negative images in an early (96–116 ms) left occipital cluster, which then extended to bilateral occipito-temporal and parietal regions in a mid-latency (160–228 ms) time interval (Schupp et al., 2003a, 2006, 2007; Carretié et al., 2004; Liddell et al., 2004; Balconi, 2006; Pegna et al., 2011; Qian et al., 2012). Importantly, and in line with previous research (Liddell et al., 2004), these rather early effects were not modulated by visual awareness. This finding supports the claim that early and mid-latency emotion effects are likely to reflect enhanced perceptual processing of and automatic attention orienting to highly motivationally relevant stimuli (Junghöfer et al., 2001; Olofsson et al., 2008; Carlson and Reinke, 2010; Keuper et al., 2013, 2014). The revealed neural patterns support a feedforward mechanism whereby enhanced automatic processing of negative stimuli begins early in the visual areas and is fed forward to higher cortical regions (Junghöfer et al., 2001, 2006; Olofsson et al., 2008; Pessoa and Adolphs, 2010). Moreover, as expected (Liddell et al., 2004; Williams et al., 2004; Kiss and Eimer, 2008; Nakajima et al., 2015) we found an interaction of exposure time and valence, which was most pronounced in the late time window (300–380 ms). Interestingly, this interaction was elicited by dorsolateral prefrontal structures and peaked in the same rDLPFC area, that was later stimulated – i.e., the region below electrode F4. In particular, this area was *less* activated in response to supraliminal negative compared to neutral stimuli, while such negative-neutral differentiation was absent in the subliminal condition. In their ERP study, Liddell et al. (2004) also reported a differentiation of amplitudes to negative compared to neutral supraliminal but not subliminal faces at similar latencies. Specifically, they found stronger positive amplitudes to negative supraliminal faces at frontocentral sites (Cz, Fz). Both, the topography and latency of this effect can nicely be reconciled with the literature of the so-called late positive potential (LPP), an emotion-sensitive ERP component indexing stronger evaluative processes in response to emotionally salient compared to neutral material (e.g., Schupp et al., 2006; Olofsson et al., 2008). However, as Liddell et al. (2004) did not employ source reconstruction approaches, the neuronal generators were not revealed in this particular study and thus remained speculative. Yet, there is ample evidence that distributed neuronal sources in visual processing areas and frontal regions contribute to the LPP (Olofsson et al., 2008; Wessing et al., 2016). Several authors have argued that stronger late-latency brain activation in visual processing areas reflects an ongoing, increasingly elaborate perceptual evaluation of emotional stimuli based on reentrant processing (Schupp et al., 2004a; Wessing et al., 2016). The strength of this effect can be modulated by several factors including the use of voluntary regulatory strategies (e.g., reappraisal, Hajcak and Nieuwenhuis, 2006), as well as rDLPFC inhibition and excitation (Notzon et al., 2017). In passive viewing tasks, where an active regulation of emotional material via prefrontal structures is not instructed, stronger emotion effects in visual processing areas can temporally co-occur with *reduced* activation to emotional material in frontal regions (Wessing et al., 2016). Thus, overall, the direction and localization of the observed interaction of valence and exposure is in line with previous findings. The absence of a contribution of visual processing areas in this effect might be a consequence of the employed back-ward masking design, in which the masking stimulus may disrupt reentrant processing of the initial stimulus in perception-related brain regions (e.g., Fahrenfort et al., 2009).

Overall, our Pre-cTBS analyses on the one hand substantiate shared emotion-sensitive feedforward mechanisms for supraliminal and subliminal perception. On the other hand, they suggest differential late regulatory mechanisms for subliminal and supraliminal affective processing. These findings fit in well with two-stage models of stimulus perception (see Schupp et al., 2006), which link early and mid-latency emotion-sensitive components (<300 ms) to a large-capacity “perceptual scanning stage providing a more or less complete analysis of sensory information” (p. 47) and propose that a conscious representation of stimuli might depend on access to a capacity-limited second stage of processing, which is likely indexed by late components (>300 ms), especially the LPP.

The right dorsolateral prefrontal localization of the late interaction of exposure time and valence further stimulates the key question of this study: How does the rDLPFC control affective processing in presence versus absence of visual awareness?

### Effects of rDLPFC Inhibition on Behavioral Measures

In the following, we will discuss the results of our behavioral measures, which were designed to test the impact of rDLPFC inhibition via cTBS on (1) affective state, (2) attention orienting to and/or attention disengagement from negative compared to neutral stimuli, (3) valence and arousal ratings, and (4) emotion discrimination.

We found no effects of rDLPFC inhibition compared to Active Control on self-reported affective state. This supports previous literature, which failed to find mood effects following a single session of inhibitory cTBS on the rDLPFC (Tupak et al., 2013). Notably, changes in self-reported mood have been reported, when the left DLPFC was targeted (Tupak et al., 2013), although not consistently (Mosimann et al., 2000; Leyman et al., 2009). Importantly, there is ample evidence for antidepressant effects of rTMS after repeated sessions of prefrontal neurostimulation: In line with theories on hemispheric asymmetry (e.g., Davidson, 1992a,b), inhibitory rTMS to the rDLPFC as well as excitatory rTMS to the left DLPFC appear to improve depressive symptoms (e.g., Fitzgerald and Daskalakis, 2011).

Despite the lack of mood effects, our study found partial support for the hypothesis of reduced negative-processing biases following rDLPFC inhibition. First, in line with van Honk et al. (2002), our findings reveal reduced emotional interference by negative facial expressions in the Gender Identification Task. Specifically, we found fewer correct responses for fearful compared to neutral faces. Such effects of emotional interference are well documented (Phaf and Kan, 2007). They reveal that it is more difficult to ignore (task-irrelevant) emotional compared to neutral material, as emotional materials capture additional attentional resources (van Honk et al., 2002). Importantly, although rDLPFC inhibition compared to Active Control stimulation did not differentially modulate interference effects on the level of accuracy, individuals in the rDLPFC inhibition condition showed less emotional interference in the reaction times. Specifically, when identifying gender for faces with fearful expressions they showed faster responses than the Active Control group, while no group differences were found in response to neutral faces. This result is in line with the observation that individuals receiving inhibitory rTMS to the rDLPFC were quicker to identify the ink-color (green, blue, red, and yellow) of supraliminal fearful faces (van Honk et al., 2002, but see Tupak et al., 2013) than those receiving sham stimulation. Overall, relatively better task-performances in such implicit tasks are thought to result from more efficient processes of attention disengagement from the (task-irrelevant) negative content of stimuli (van Honk et al., 2002; Koster et al., 2005; Sagliano et al., 2016).

Second, in addition to more effective attentional disengagement from negative stimuli, the rDLPFC inhibition group rated negative scenes as less negative and less arousing. As predicted by theories of hemispheric asymmetries (for reviews, see Davidson, 1992a,b; Harmon-Jones et al., 2010; Grimshaw and Carmel, 2014), this finding further substantiates that rDLPFC inhibition may reduce withdrawal-related behaviors as indexed by the observed attenuation of negativity biases.

However, reductions of negativity biases following rDLPFC inhibition were not consistently observed in all tasks. We were not able to replicate effects of neurostimulation on the identification of fearful versus neutral faces following rDLPFC inhibition (Zwanzger et al., 2014). This lack of effect might result from several disparities between these two studies. First, we tested Chinese, not German participants, which may have affected task performance during the Identification of Facial Expressions of Caucasian faces in this task. Second, the frequent repetition of facial stimuli in the study by Zwanzger et al. (2014) may have influenced effects of rDLPFC inhibition on facial expression identification. In particular, Zwanzger et al. (2014) repeated the Facial Expression and Gender Identification Task before and after rTMS application, and used the same faces during the intermediate passive viewing task. By contrast, we administered the Facial Expression and Gender Identification Task only once and presented participants with subliminal and supraliminal negative and neutral scenes in the preceding passive viewing task. A third explanation for discrepancies in the findings may result from the use of different stimulation protocols [inhibitory low-frequency rTMS (1 Hz) vs. inhibitory cTBS] and control conditions (Sham vs. Active Control).

Further, the visual awareness task yielded no effects of cTBS stimulation. Neither in the subliminal nor in the supraliminal condition did we find evidence for an effect of rDLPFC inhibition. Thus, it seems unlikely that rDLPFC inhibition affected the identification and/or discrimination of negative and neutral stimuli. Importantly, this result confirms that in both groups, emotional contents of subliminal images were successfully rendered unrecognizable. This should be taken into consideration, when interpreting effects of rDLPFC inhibition on neurophysiological findings.

Taken together, our behavioral findings support the notion of a causal role of frontal structures in the regulation of negative stimulus processing (Phan et al., 2005). Yet, reductions of negativity biases following rDLPFC inhibition were observed in some tasks, while they were absent in others and did not readily translate into mood changes. In combination with inconsistent findings in the literature, this suggests that effects of rDLPFC inhibition on behavior might depend on various boundary conditions (e.g., rTMS protocol, frequency and site of stimulation, familiarity with stimulus material, etc.), that require further future investigation.

### Effects of rDLPFC Inhibition on Neurophysiological Measures

Importantly, observed reductions of behavioral negativity biases may result from different neuronal mechanisms. Theoretically, they may on the one hand be due to *reduced automatic encoding of negative material in the feedforward pathway*, contributing to less interference by and reduced negativity and arousal ratings of stimuli. On the other hand, they may be due to *increased speed and/or efficiency of encoding and projection of negative cues* to frontal regions and/or *enhanced prefrontal control at later processing stages*. In the following, we will closely evaluate these interpretations based on the time-course rTMS-driven emotion effects.

#### Awareness-Independent Effects of rDLPFC Inhibition: Automatic Valence Processing

On a neurophysiological level, rDLPFC inhibition relatively enhanced occipito-parietal and centro-parietal brain activity for both subliminal and supraliminal negative images. These effects started in early intervals and were strongest at mid-latency and late processing stages (112–268 ms; 320–380 ms). The early onset of these effects (<120 ms) as well as their localization in perception-related brain areas substantiate previous reports of prefrontal modulatory influence on early brain signatures of emotional attention (also see Zwanzger et al., 2014; Notzon et al., 2017). Perception-related brain areas including occipito-parietal and also frontal regions have been previously implicated in stimulus-driven mechanisms of emotional attention (Olofsson et al., 2008) and the automatic feedforward sweep of negative information toward higher cortical regions (Schupp et al., 2006; Pessoa and Adolphs, 2010; Zwanzger et al., 2014). Importantly, these rather early interactions between stimulation site and valence were not affected by exposure time, which implies that even highly automatic stimulus-driven processes can be under prefrontal control. In line with this, Corbetta and Shulman (2002) proposed that interactions between (automatic) bottom-up processing and top-down control may work together to guide attention mechanisms. Specifically, it was proposed that “... task-relevant signals from the dorsal system ‘filter’ stimulus-driven signals in the ventral system, whereas stimulus-driven ‘circuit-breaking’ signals from the ventral system provide an interrupt to the dorsal system, reorienting it toward salient stimuli” (Fox et al., 2006). In line with our neurophysiological findings, this model predicts that inhibition of the rDLPFC as part of the dorsal system should reduce top-down control and thereby *enhance* bottom-up processing of salient (here: negative) stimuli in perception-associated brain areas (see also Zwanzger et al., 2014; Notzon et al., 2017).

However, previous research also suggests that strong stimulus-driven emotional responses are typically associated with *enhanced* interference effects on the behavioral level. Thus, predictions of the model by Corbetta and Shulman (2002) as well as our neurophysiological findings on cTBS effects on automatic valence processing seem to contradict our behavioral data, which – by contrast – suggest *reduced* interference by negative information. How can *boosted (early) brain activity reflecting enhanced emotional attention* be reconciled with *reduced behavioral negativity biases*? One possibility may be the initiation of *a (compensatory) top-down regulatory mechanism* that contributes to the ultimately observed behavioral effects. If such mechanism exists, one would expect the recruitment of frontal brain areas that support later, more elaborate awareness-dependent processes of emotion regulation (Kozel and George, 2002; van Honk et al., 2002; Bishop et al., 2004; Siegle et al., 2007; Poldrack et al., 2008; Guse et al., 2010; Ochsner et al., 2012; Ironside et al., 2016).

#### Awareness-Dependent Effects of rDLPFC Inhibition: Elaborate Valence Processing

In fact, our findings indicate that rDLPFC inhibition enhances relatively late (292–360 ms) brain activity exclusively in response to negative images that are available to visual awareness, while reduced brain activity for negative compared to neutral images was found in the subliminal condition and in the Active Control group. Of note, to our knowledge, this is the first available evidence for a differential influence of rDLPFC inhibition on spatiotemporal neural correlates of subliminal vs. supraliminal negative processing. This interactive effect was found in bilateral dorsolateral prefrontal frontal regions. Compared to the interaction of valence and exposure in the Pre-cTBS data, which – interestingly – was found in the same brain region that was afterward stimulated (below electrode F4), this effect peaked in more ventral parts of the bilateral DLPFC.

Following Fox et al. (2006) this interactive effects might be the result of stronger bottom-up stimulus-driven “circuit-breaking” signals elicited by negative stimuli in the rDLPFC inhibition group, which then interrupt dorsal system functioning. Our findings indicate that this interruption and a reorientation of the dorsal system toward salient stimuli only takes place if stimuli enter visual awareness. In the light of our behavioral findings, which show a reduction of negative-processing biases following rDLPFC inhibition and previous studies associating inhibitory, emotion-regulatory processes with DLPFC functioning (van Honk et al., 2002; Ochsner et al., 2012), one might speculate that this stronger prefrontal activation to negative supraliminal stimuli reflects an enhanced awareness-dependent down-regulation of negative scene processing. Such mechanism eventually leads to facilitated disengagement from and less negative and less arousing evaluations of negative stimuli.

Yet, although this idea aligns well with our behavioral findings and the previous literature, it appears surprising that such mechanism is seemingly underpinned by brain activity adjacent to and partly overlapping with the (inhibited!) rDLPFC. How can a brain region that was inhibited by means of cTBS effectively support emotion-regulatory functions? To account for this, it seems necessary to compare the exact localization of the stimulated brain region (i.e., those parts of the rDLPFC that are located directly under F4) with the observed cluster of the three-way interaction of valence, exposure, and stimulation. In fact, the stimulated area is localized slightly more dorsal than the observed effect. Although highly speculative, this might suggest that different parts of the rDLPFC serve different types of emotional control processes. First, the more dorsal part below F4 might control automatic bottom-up processes of emotional attention early in the processing stream and independently of visual awareness. Second, bilateral, more ventral parts of the DLPFC may support later more elaborate regulatory mechanisms that depend on visual awareness. Overall, our findings suggest that that behavioral responses to emotional stimuli depend on the flexible interplay of mechanisms that support fast automatic responses to emotional stimuli on the one hand and subsequent (compensatory?) regulatory strategies on the other.

### Limitations

Overall, our findings indicate that the spatiotemporal interplay between feedforward pathways in occipito-parietal areas and prefrontal regions, as well as interactions of different prefrontal brain regions underpin distinct aspects of affective processing. In the light of this complex interplay, the choice of the vertex (electrode Cz) as the Active Control stimulation site requires critical reflection. We based our decision for this use of Cz on several considerations: First, compared to passive-controlled designs using “Sham” stimulation (e.g., Zwanzger et al., 2014), active-controlled designs enable more specific conclusions regarding the specific contribution of the stimulated brain region to the observed effects. We were able to replicate early enhanced bottom-up processing of negative stimuli following rDLPFC inhibition (Zwanzger et al., 2014) when using Cz as an Active Control site. This strengthens the conclusion that emotion perception is in fact controlled by rDLPFC function. However, as an inherent disadvantage of active-controlled designs, an additional contribution of the Active Control site to this effect cannot be ruled out. Second, our aim to compare subliminal with supraliminal perceptual processing of emotional stimuli required an Active Control site with a minor role in earliest stages of visual feedforward-processing. Based on studies which localized early and also mid-latency responses to negative affective (vs. neutral) visual stimuli mainly in occipital and temporal regions (Junghöfer et al., 2001, 2006; Olofsson et al., 2008; Bayle et al., 2009), and also based on a recent meta-analysis of 157 fMRI studies on emotional face and emotional scene processing, which did not reveal significant affect-modulated activation of central structures (Sabatinelli et al., 2011), we selected Cz as a control site. However, as can be seen in **Figure 2**, our Pre-cTBS data clearly show a contribution of parietal brain regions (below electrode Cz) in mid-latency emotion effects. Further, especially late emotion-sensitive ERP components are often visible at centro-parietal midline electrodes (e.g., Schupp et al., 2004b). Although the estimated underlying sources of these late ERP components were mainly found at visual sensory and parietal and not central regions (Sabatinelli et al., 2006), we nevertheless cannot exclude the possibility that stimulation of Cz – e.g., by co-stimulation of parietal structures – influenced our results, in particular with regard to the awareness-dependent effects observed at later processing stages. Therefore, it would be of great interest to replicate this study under varying control conditions, e.g., passive “Sham” stimulation (e.g., Zwanzger et al., 2014). It would also be of interest to further investigate the hemispheric specificity of DLPFC inhibition on the interplay of valence processing and awareness, for example by targeting the left DLPFC. Future studies, which aim at elucidating the specific functional contribution of different brain regions and their functional connectivity may help to better understand how affective information is processed over time – with and without visual awareness.

Importantly, and in the light of these limitations, neuronal and behavioral findings of our study suggest that effective down-regulatory mechanisms following rDLPFC inhibition via cTBS may exclusively apply to negative stimuli that reach participants awareness. On the other hand, neurophysiological findings indicated that both supra- and subliminal negative stimuli received enhanced emotional attention following rDLPFC inhibition. Together, these findings imply that emotional stimuli that remain below awareness might still influence affective states in a subtler way. Such type of emotional reactivity might be difficult to capture by traditional behavioral tasks. Although we included several behavioral tasks to explore the direction that rTMS effects would take on different aspects of emotion processing, one additional limitation of our study regards the interpretability of our neuronal effects. As the neural data were collected during a passive viewing task, which required no responses from participants, the association between our electrophysiological and behavioral results remains partially speculative. Further, with exception of the visual awareness task, all behavioral measures used supraliminal images. This prevented firm conclusions on rTMS-induced mechanisms that are specific to subliminal affective processing. Therefore, future studies should employ active-response tasks to differentiate neurocognitive mechanisms involved in subliminal and supraliminal affective processing and their modulation by rDLPFC stimulation.

### Implications and Future Outlook

Overall, our study has provided important insights on the causal influence of rDLPFC function on affective processing in presence and absence of visual awareness. In summary, we found evidence for reduced emotional interference by, and less negative and aroused ratings of negative supraliminal stimuli following rDLPFC inhibition. rDLPFC inhibition did not affect self-reported mood or the discrimination performance in the awareness task. Based on our finding of enhanced neurophysiological emotion effects at early and mid-latency processing stages, we suggested that rDLPFC inhibition boosts automatic processes of “emotional attention” independently of visual awareness. Further, our study revealed to our knowledge the first available evidence for a differential influence of rDLPFC inhibition on subliminal versus supraliminal neural emotion processing. We tentatively argued that this effect might reflect enhanced awareness-dependent down-regulation of negative scene processing, eventually leading to facilitated disengagement from and less negative and arousing evaluations of negative supraliminal stimuli. Future research is needed to understand in more detail how targeted non-invasive brain stimulation via rTMS may differentially influence subliminal and supraliminal emotional stimulus processing. A clearer picture of these mechanisms might have crucial implications for the understanding and treatment of mood and anxiety disorders, which are not only maintained and exacerbated by “conscious” negativity biases, but also by processes induced by subliminal emotional triggers. Bar-Haim et al. (2007) for instance revealed that for subliminally presented stimuli, anxiety patients showed a negativity bias but non-anxious individuals even revealed a bias away from threat. Therefore, studies combining neurostimulation techniques with neurophysiological and behavioral measures of conscious and preconscious affective processing not only in healthy controls, but also in mood- and anxiety-disordered patients may contribute important insights regarding the therapeutic use of rTMS to treat emotional dysfunctions and processing biases.

## Author Contributions

KK, ET, and TL designed the experiment. ET conducted the study. KK and ET analyzed the data and wrote the manuscript. MJ and CC provided technical support during data collection and analysis, respectively. All authors read the manuscript and provided feedback.

## Conflict of Interest Statement

The authors declare that the research was conducted in the absence of any commercial or financial relationships that could be construed as a potential conflict of interest.

## Footnotes

1 http://www.magstim.com/

2 http://www.mathworks.com/

3 https://www.pstnet.com/

4 compumedicsneuroscan.com
